# Cell surface ectodomain integrity of a subset of functional HIV-1 envelopes is dependent on a conserved hydrophilic domain containing region in their C-terminal tail

**DOI:** 10.1186/s12977-018-0431-4

**Published:** 2018-07-20

**Authors:** Sweety Samal, Supratik Das, Saikat Boliar, Huma Qureshi, Tripti Shrivastava, Naresh Kumar, Sandeep Goswami, Manish Bansal, Bimal K. Chakrabarti

**Affiliations:** 1 THSTI-IAVI HIV Vaccine Design Program, Translational Health Science and Technology Institute, NCR Biotech Science Cluster, 3rd Milestone, Faridabad-Gurgaon Expressway, P.O. Box # 04, Faridabad, Haryana 1221001 India; 2 IAVI Neutralizing Antibody Center, The Scripps Research Institute, La Jolla, CA USA; 3 Innovation & Translation, ABL, Inc., 9800 Medical Center Drive, Building D, Rockville, MD 20850 USA

## Abstract

**Background:**

HIV-1 Env gp160 is cleaved to form gp120 and gp41 and the functional HIV-1 Env is a trimer of non-covalently associated heterodimeric subunits, gp120 and gp41. The cleaved, native, trimeric form of Envs expose only broadly neutralizing antibody (bNAb) epitopes while occluding epitopes targeted by non-neutralizing antibodies (non-NAbs). We and others have previously observed that efficient cleavage of Envs into their constituent subunits co-relates with specific binding to bNAbs and poor binding to non-neutralizing antibodies (non-NAbs). Such Envs have been identified from clades A, B and C which make up a majority of globally circulating HIV-1 strains. Frequently, the C-terminal tail (CT) of Envs is deleted to enhance expression and stabilize soluble Env-based vaccine immunogens. Deletion of CT of efficiently cleaved Indian clade C Env 4-2.J41 results in recognition by both NAbs and non-NAbs. It is to be noted that uncleaved Envs bind to both NAbs and non-NAbs. So we investigated whether altered antigenicity upon CT deletion of efficiently cleaved Envs is due to inefficient cleavage or conformational change as the mechanism by which the CT regulates the ectodomain (ET) integrity is not well understood.

**Results:**

We studied the effect of CT deletion in four membrane bound efficiently cleaved Envs, A5 (clade A), 4-2.J41 (clade C), JRFL and JRCSF (clade B). Deletion of CT of the Envs, JRCSF and 4-2.J41, but not JRFL and A5 alter their ET antigenicity/conformation without affecting the cleavage efficiency. We carried out a series of deletion mutation in order to determine the region of the CT required for restoring native-like antigenicity/conformation of the ET of 4-2.J41 and JRCSF. Extending the CT up to aa753 in 4-2.J41 and aa759 in JRCSF, which includes a conserved hydrophilic domain (CHD), restores native-like conformation of these Envs on the plasma membrane. However, CT-deletion in 4-2.J41 and JRCSF at the pseudovirus level has either no or only modest effect on neutralization potency.

**Conclusion:**

Here, we report that the CHD in the CT of Env plays an important role in regulating the ET integrity of a subset of efficiently cleaved, functional Envs on the cell surface.

**Electronic supplementary material:**

The online version of this article (10.1186/s12977-018-0431-4) contains supplementary material, which is available to authorized users.

## Background

The functional HIV-1 Env is a trimer of a non-covalently associated heterodimeric protein (gp120 and gp41 subunits), which mediates the entry of the virus into the host cell by interacting with cellular receptors [1–3]. The cleaved, native, trimeric form of Envs expose only broadly neutralizing antibody (bNAb) epitopes while occluding the epitopes targeted by non-neutralizing antibodies (non-NAbs) [4–7]. Since uncleaved and non-native forms of Env bind to non-NAbs [6–8] and may aid in immune evasion during HIV-1 infection by eliciting non-NAbs [9, 10], the current focus for designing immunogens is based on generating native, cleaved forms of Envs [6, 11, 12]. The only reported uncleaved, soluble Envs, that display exclusively bNAb epitopes, are the artificially generated single chain trimers [13–15]. However, in order to use Envs as immunogens for DNA, viral vector, virus-like particle based vaccination studies, it is essential that they are efficiently cleaved and display only broadly neutralizing epitopes on the cell surface. Furthermore, priming with Env expressing DNA followed by protein boosting has been shown to elicit more durable and qualitatively superior antibody response than protein alone [16–18]. Therefore, it is important that Envs need to display specifically bNAb epitopes on the cell surface when they are delivered through DNA. The American clade B Envs, JRFL and JRCSF, Indian clade C Env, 4-2.J41, and African clade A Envs, BG505 and A5 [19] are naturally occurring, efficiently cleaved Envs, when expressed on cell membrane and all of them bind exclusively to bNAbs [8, 20, 21]. Envs belonging to clades A, B and C make up about 75% of globally circulating HIV-1 strains.

The about 150 amino acid long CT (cytoplasmic tail) of Env contains several motifs [22–24] that are highly conserved across different HIV-1 clades and plays a critical role in modulating multiple Env functions [25, 26]. The cytoplasmic tail contains the tyrosine (YXXϕ) and di-leucine (LL) motifs, palmitoylated conserved cysteines, lentivirus lytic peptides (LLP), calmodulin binding domains [22–24]. The YXXΦ (where X can be any amino acid, and Φ is a bulky, hydrophobic group) motif is shown to be crucial for their endocytosis following internalization of Env; thus disruption of this motif increases Env surface expression [25, 27]. Therefore, CT of Env is regularly deleted in order to enhance expression [28] on cell surface and also to stabilize the soluble versions (SOSIP and NFL) of native-like trimeric Env immunogens [11, 14]. Thus, from a HIV-1 vaccine immunogen development point of view, it is important to understand the effect of CT truncation on antigenic properties of Env. Truncation of the CT affects ectodomain (ET) integrity of Envs but the cleavage status of these Envs is unknown [29–31]. In a recent report [32], using partially cleaved Envs, it has been shown that truncation of the C-terminus of these Envs expose non-neutralizing epitopes [32]. However, such studies have not been carried out in detail with naturally occurring, efficiently cleaved Envs, which are the closest approximations of functional HIV-1 Envs. Here, we show that naturally occurring, efficiently cleaved membrane-bound forms of Envs show phenotypic diversity in their ectodomain (ET) when their C-terminal tails are deleted without altering cleavage efficiency. C-terminally truncated JRFL and A5 Envs are able to maintain their antigenicity and conformation i.e. they do not expose non-neutralizing epitopes, but CT-deleted JRCSF and 4-2.J41 exposes non-neutralizing epitopes. Furthermore, CT deletion in all four Envs does not alter their cleavability. A conserved hydrophilic domain present in the CT of 4-2.J41ΔCT_753_ and JRCSFΔCT_759_ Envs restores native-like ET conformation similar to that of the wild type protein. CT-deletion in 4-2.J41 Env at the level of virus leads to modest resistance to conformational and trimer-selective bNAbs and sensitivity is not restored by restoring the conserved hydrophilic domain at the C-terminus. Furthermore, 4-2.J41ΔCT pseudoviruses become more sensitive to non-neutralizing antibodies at lower temperature and resistance is not restored by restoring the conserved hydrophilic domain. In addition, JRCSFΔCT Env pseudotyped viruses show similar neutralization potency for bNAbs, except 10E8. The sensitivity of JRCSFΔCT Env pseudotyped viruses to non-NAbs is similar to as it is seen for the virus pseudotyped with full length JRCSF Env. Taken together our findings demonstrate for the first time that conformational and antigenic integrity of the ectodomain of a subset of efficiently cleaved, functional Envs on the plasma membrane is dependent on the conserved hydrophilic domain present in their C-terminal tail. The significance of these findings on HIV-1 Env-based candidate vaccine development and the biology of these Envs are discussed.

## Results

### C terminal tail deletion of 4-2.J41 affects ectodomain conformation/antigenicity

**Fig. 1:**
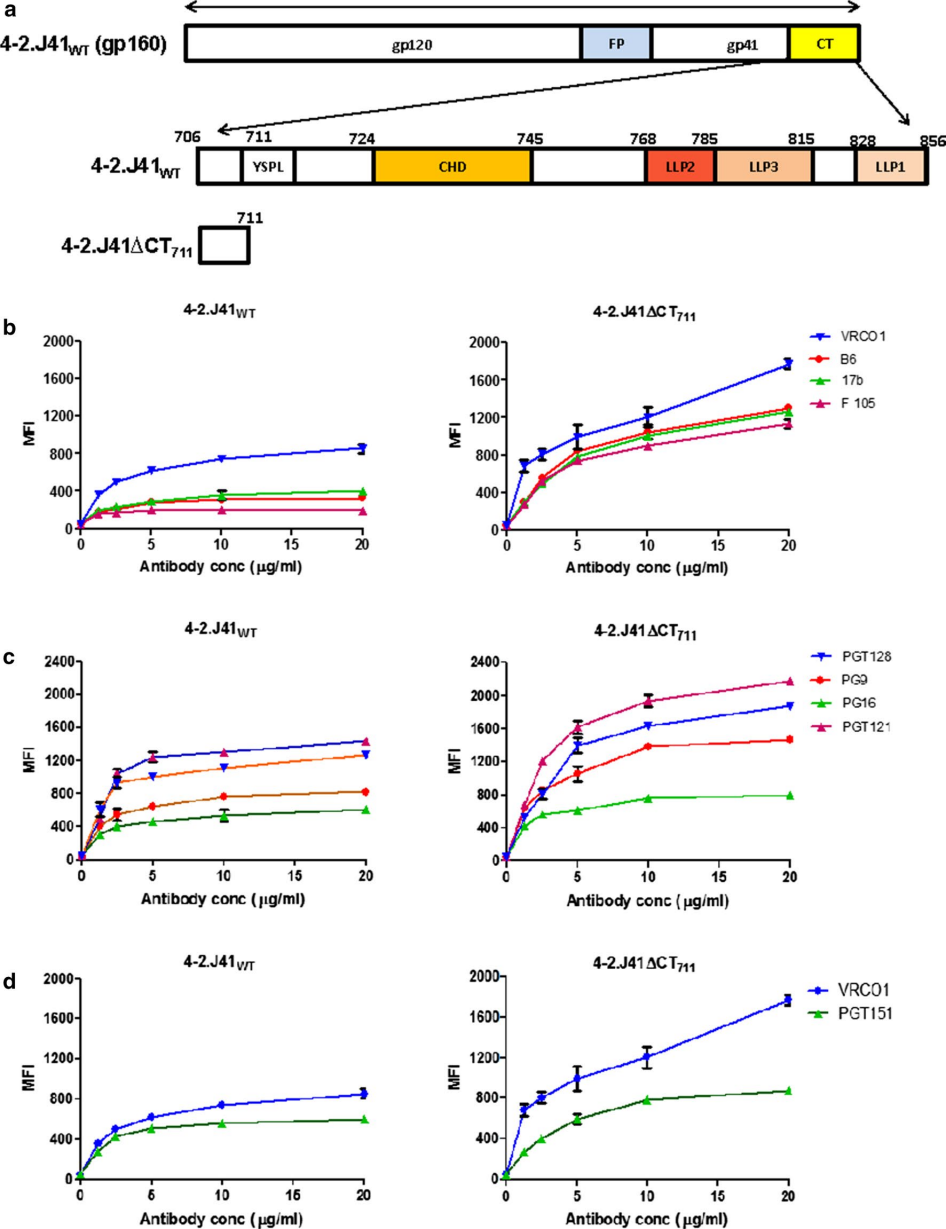
CT-deleted 4-2.J41 Env has altered cell surface antigenicity/conformation. **a** Schematic of different domains in the C-terminal tail of HIV-1 Env. **b** FACS based cell surface staining assays of 4-2.J41_WT_ and 4-2.J41ΔCT_711_ with cleavage non-specific bNAb, VRC01 and non-NAbs, F105, 17b and b6 over a range of antibody concentrations. **c** FACS based cell surface staining assays of 4-2.J41_WT_ and 4-2.J41ΔCT_711_ with glycan-dependent, conformational bNAbs, PG9, PG16, PGT121, PGT128. **d** FACS based cell surface staining assays of 4-2.J41_WT_ and 4-2.J41ΔCT_711_ with bNAb, VRC01 and trimer-selective, cleavage-specific bNAb, PGT151

It has been reported previously that truncation of the C-terminus of HIV-1 Envs generally leads to higher expression but affects their antigenicity, which is evident from exposure of epitopes for non-neutralizing antibodies [28–32]. These studies were primarily carried out with either partially cleaved Envs, or the cleavage efficiency of those Envs was not reported [30, 32]. However, studies with such Envs do not give a true representation of the structure–function relationship of the CT of functional Envs as an efficiently cleaved Env exposes only broadly neutralizing epitopes and are thus the closest representation of functional Envs, whereas uncleaved Envs bind to both neutralizing and non-neutralizing antibodies. The CT deletion in both 4-2.J41 and JRFL, have only modest effect on neutralization potency of pseudotyped viruses by bNAbs while they remain resistant to neutralization by non-NAbs [5, 20]. However, deletion of the C-terminus of the Env, 4-2.J41 increases expression, but exposes non-neutralizing epitopes on the cell surface [20] suggesting that the effect of C-terminal truncation is different between Envs expressed on cell surface and on viral membrane. We, therefore, investigated the effect of C-terminal deletion on the cell surface expression, conformation and antigenicity of 4-2.J41 Env and determined the ratio of binding to bNAbs versus non-neutralizing antibodies (non-NAbs). As shown in Fig. 1a, C-terminal deletion up to position 711 eliminates all the major known domains of the CT of Env. Deletion of the C-terminus resulted in higher expression of 4-2.J41ΔCT compared to wild type 4-2.J41 as the cleavage-independent bNAb VRC01 showed much higher binding to the ΔCT form as compared to wild-type (Fig. 1b) and we also observed enhanced binding to the non-NAbs F105, b6 and 17b (Fig. 1b). We determined the ratio of binding to different non-NAbs in comparison to VRC01 at 20 μg/ml antibody concentration (Additional file 1: Table S1a). We find that the ratio of binding to non-NAbs F105, b6 and 17b versus VRC01 changes from 0.22, 0.38, 0.47 for wild type 4-2.J41 to 0.64, 0.73, 0.71 for 4-2.J41ΔCT (Additional file 1: Table S1a), respectively, suggesting that the non-neutralizing epitopes become exposed in 4-2.J41ΔCT. We next tested the binding of 4-2.J41 and 4-2.J41ΔCT to the glycan-dependent, conformational bNAbs PG9, PG16, PGT121 and PGT128 in comparison to VRC01 and determined their binding ratios (Fig. 1c and Additional file 1: Table 1b). The ratio of binding to PG9 versus VRC01 did not change between 4-2.J41 and 4-2.J41ΔCT but binding to PG16, PGT121 and PGT128 showed a decline from 0.7, 1.68, 1.48 for 4-2.J41 to 0.44, 1.22, 1.05, respectively, for 4-2.J41ΔCT (Fig. 1c and Additional file 1: Table S1b) suggesting that the conformation of 4-2.J41 is affected upon C-terminal tail deletion. Next, we tested 4-2.J41 and 4-2.J41ΔCT for their ability to bind to the trimer-selective, cleavage-specific bNAb PGT151 in comparison to VRC01 and determined the ratio of binding (Fig. 1d and Additional file 1: Table S1c). The binding ratio fell from 0.71 for 4-2.J41 to 0.33 for 4-2.J41ΔCT (Additional file 1: Table S1c) suggesting that the native conformation of 4-2.J41 is affected upon CT deletion. Taken together these studies suggest that deletion of the C-terminal tail of 4-2.J41 cause conformational changes in 4-2.J41ΔCT and exposes non-neutralizing epitopes.

### C-terminal tail deleted 4-2.J41 is efficiently cleaved

**Fig. 2:**
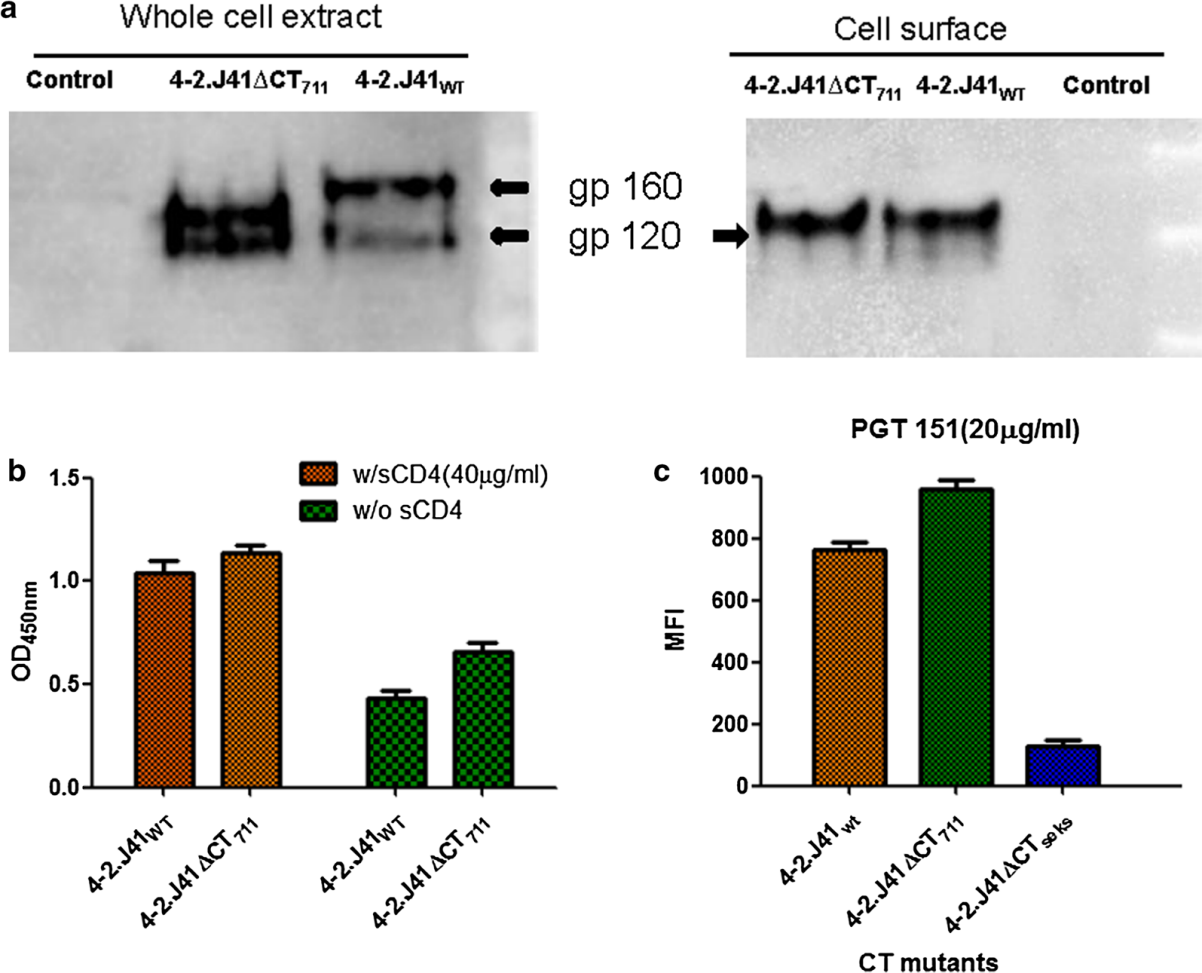
CT-deleted 4-2.J41 Env is cleaved. **a** Western blot analysis of cell lysates (left panel) and neutravidin-agarose precipitates (right panel) of cell surface biotinylated mock transfected, 4-2.J41_WT_ and 4-2.J41ΔCT_711_ Env transfected 293T cell lysates are shown. **b** Spontaneous and sCD4-induced gp120 shedding of 4-2.J41_WT_ and 4-2.J41ΔCT_711_ Env transfected 293T cells incubated separately with 20 ug/ml of sCD4 determined by ELISA. **c** FACS-based binding of 4-2.J41_WT_ and 4-2.J41ΔCT_711_ on cell surface to trimer-selective, cleavage-specific PGT151 bNAb. The Env, 4-2.J41_SEKS_, a cleavage defective, non-native form of 4-2.J41 Env was used as a negative control. The error bars indicate the standard mean obtained from three independent experiments performed in duplicates, *P* < 0.0001

We next investigated whether the observed alteration in conformation/antigenicity of 4-2.J41ΔCT was due to inefficient cleavage of the ΔCT version as uncleaved Envs bind to non-NAbs [8, 20, 21, 33]. We carried out neutravidin-agarose pull down of biotinylated cell surface proteins from 4-2.J41_WT_ and 4-2.J41ΔCT_711_ transfected cells and then probed the precipitate in western blots with anti-clade C antibodies (Fig. 2a). The crude whole cell extract showed two distinct bands corresponding to gp120 and the uncleaved gp140/gp160 (4-2.J41ΔCT_711_/4-2.J41_WT_) (Fig. 2a, left panel). The neutravidin-agarose precipitates of both 4-2.J41_WT_ and 4-2.J41ΔCT_711_ extracts showed a single band corresponding to gp120 (Fig. 2a, right panel) suggesting that like 4-2.J41_WT_ the CT deleted 4-2.J41, 4-2.J41ΔCT_711_ is efficiently cleaved on the cell surface. We further confirmed efficient cleavage of 4-2.J41ΔCT_711_ by gp120 shedding assay in the absence and presence of sCD4 (Fig. 2b). We found by both western blot analysis (data not shown) and ELISA assay (Fig. 2b) that both 4-2.J41_WT_ and 4-2.J41ΔCT_711_ proportionately shed gp120 and this shedding is enhanced upon incubation with sCD4 at the concentration of 40 ug/ml (Fig. 2b). Finally we looked at binding to the trimer-selective, cleavage-specific bNAb PGT151 at 20 µg/ml concentration by FACS-based cell surface antibody binding assay (Fig. 2c). We find that both 4-2.J41_WT_ and 4-2.J41ΔCT_711_ bind to PGT151 efficiently while the uncleaved 4-2.J41_SEKS_ binds to PGT151 poorly (Fig. 2c). Taken together these studies demonstrate that the altered conformation/antigenicity of 4-2.J41ΔCT_711_ is not due to a defect in cleavage efficiency.

### LLP domains in 4-2.J41 Env CT do not restore ET conformation/antigenicity

**Fig. 3:**
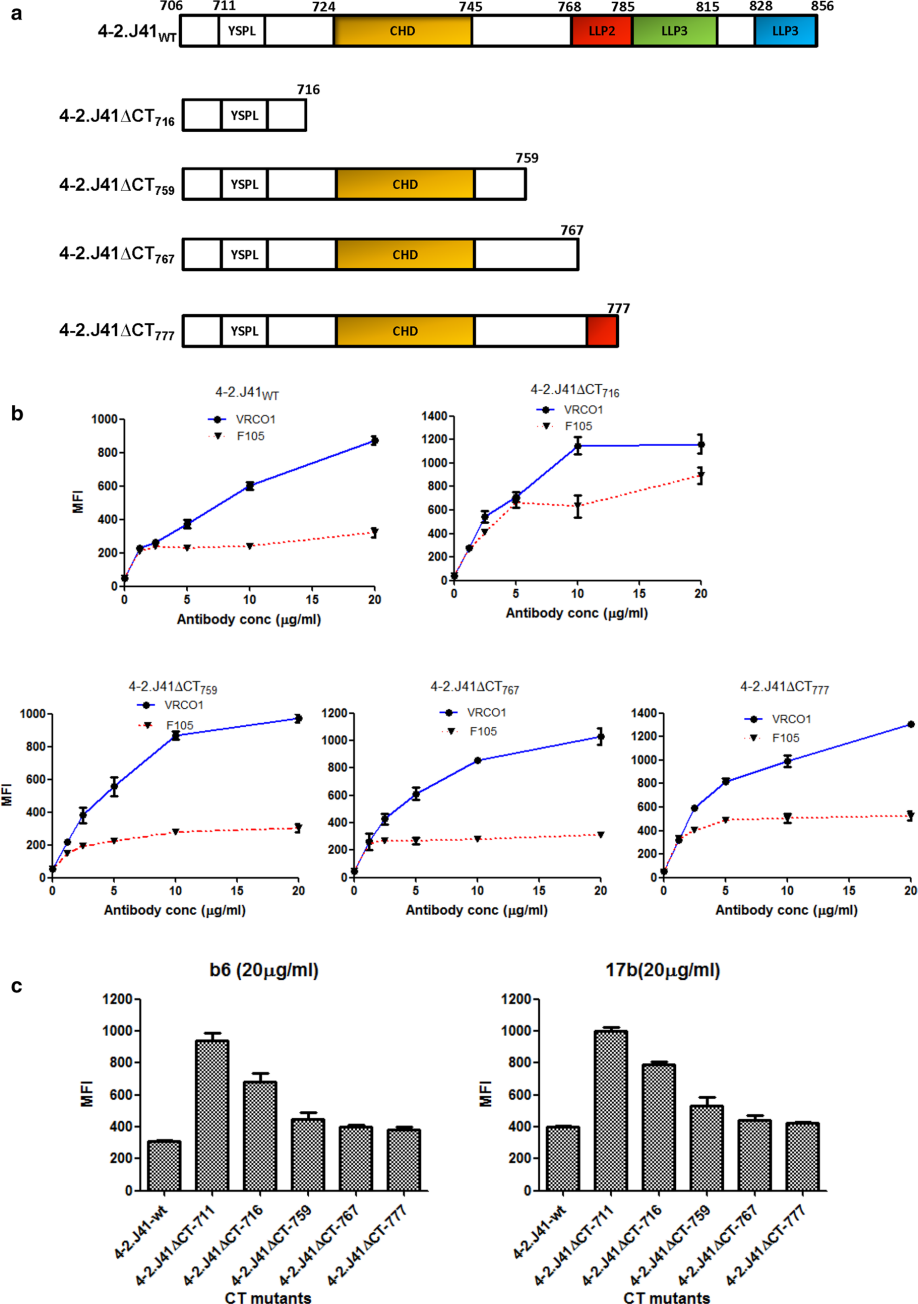
Restoration of cell surface antigenicity/conformation in CT mutants of 4-2.J41. **a** Schematic representation of LLP domain deleted mutants. **b** FACS-based cell surface staining assay of LLP domain deleted mutants with the bNAb, VRCO1 and non-NAb, F105. **c** Binding of LLP domain deleted mutants to CD4bs-directed non-NAb, b6 and CD4i-directed non-NAb, 17b at 20 µg/ml concentration. The error bars indicate the standard mean obtained from two independent experiments in duplicates. *P* < 0.0001

Having shown that 4-2.J41ΔCT_711_ Env has altered ectodomain antigenicity/conformation, we sought to examine whether any conserved domains in its CT are capable of restoring wild type antigenicity/conformation on cell surface. The highly conserved amphipathic α-helical LLP-1, LLP-2, LLP-3 domains located at the C-terminus of HIV-1 Env (CT) have been reported to play an important role in viral Env stability, oligomerization, membrane association and viral replication [34]. Hence, to investigate the importance of LLP domain of 4-2.J41_WT_ Env on antigenicity/conformation, we deleted LLP domains (LLP1: 828-856, LLP2: 768-785, LLP3: 785-815) in the CT of 4-2.J41_WT_ Env. We constructed four truncated forms of 4-2.J41_WT_ by incorporating stop codons at aa positions 717 (4-2.J41ΔCT_716_), 760 (4-2.J41ΔCT_759_), 768 (4-2.J41ΔCT_767_) and 778 (4-2.J41ΔCT_777_), respectively (Fig. 3a). It is to be noted that the amino acid at position, 716 is located prior to the conserved domains in CT. In 4-2.J41ΔCT_759_ Env, the conserved aa, Cys at position, 764 is absent, which ablates the palmitoylation site. In 4-2.J41ΔCT_767_ Env, the conserved aa Cys at 764 is present. In 4-2.J41ΔCT_777_ Env, a small part of LLP2 domain is present. Like 4-2.J41_WT_ Env, the 4-2.J41ΔCT_759_, 4-2.J41ΔCT_767_ and 4-2.J41ΔCT_777_ Env proteins expressed on cell surface showed similar level of binding to VRCO1 and only minimal binding to non-NAb, F105 (Fig. 3b). In contrast, the 4-2.J41ΔCT_716_ Env, which retains only 10 amino acids of the cytoplasmic tail, showed increased binding to non-NAb F105 relative to VRC01 (Fig. 3b). Additionally, 4-2.J41ΔCT_716_ Env showed marked increase in binding to CD4bs-directed non-NAb, b6 and CD4i-directed non-NAb, 17b (Fig. 3c). Furthermore, reduction in binding to non-NAbs, b6 and 17b was observed upon increasing the CT length beyond position 759 (Fig. 3c). Taken together, these results suggest that none of the LLP domains in 4-2.J41_WT_ Env are required for restoration of native ectodomain antigenicity/conformation.

### Conserved hydrophilic domain in 4-2.J41ΔCT_753_ restores ET conformation/antigenicity

**Fig. 4:**
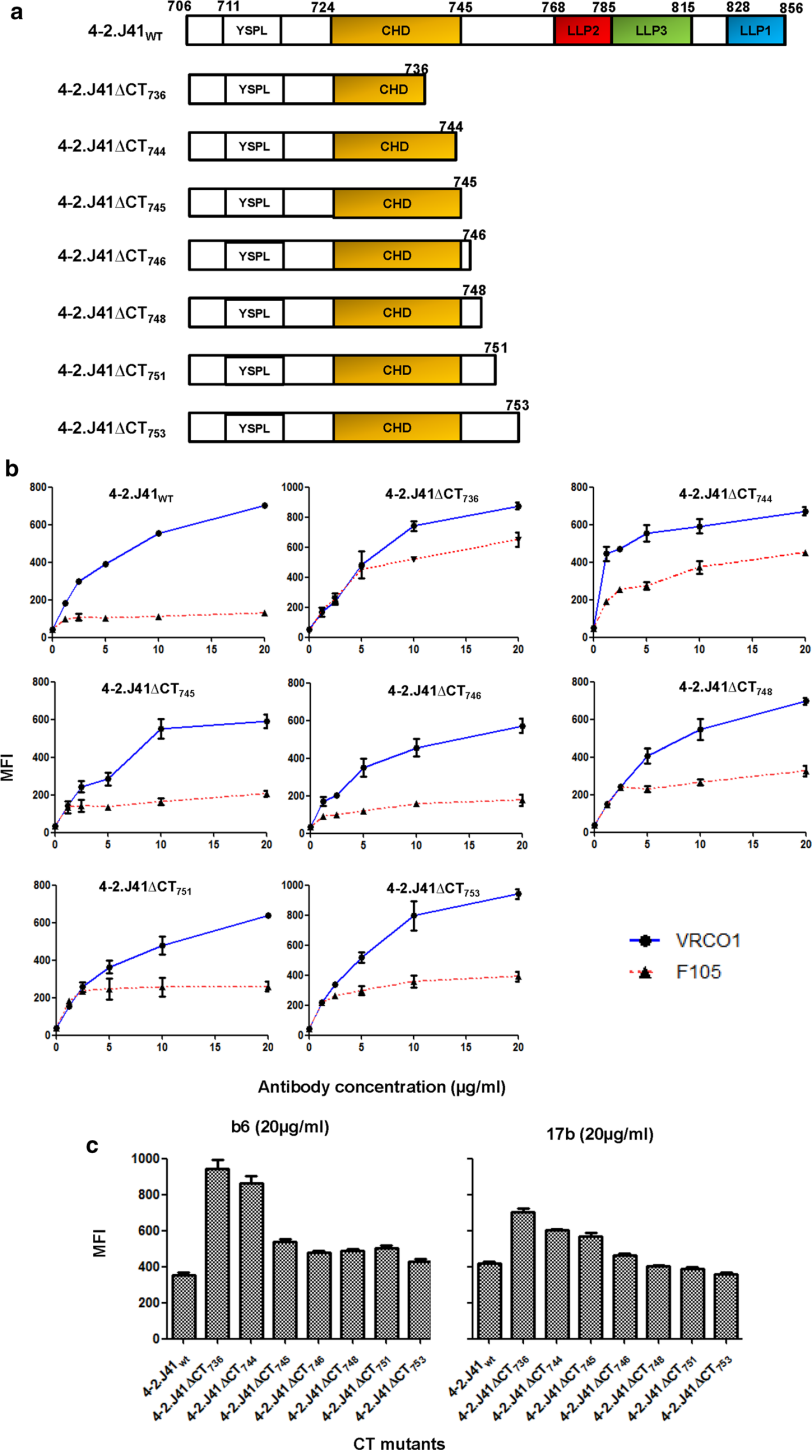
Minimal CT region of 4-2.J41wt Env required for restoring native antigenicity/conformation. **a** Schematic representation of CHD deleted mutants of 4-2.J41 Env proteins. **b** FACS-based cell surface staining assay of CHD deleted mutants with the bNAb, VRCO1 and non-NAb, F105. **c** Binding of CHD deleted mutants to CD4bs-directed non-NAb, b6 and CD4i-directed non-NAb, 17b at 20 µg/ml concentration. The error bars indicate the standard mean obtained from two independent experiments run in duplicates (*P* < 0.0001)

Sequence analysis of HIV-1 Env proteins from different clades have revealed the presence of a conserved 22 amino acid domain (residues 724-745), different from LLP, in the CT of the HIV-1 Envs [22, 24]. The HIV-1 clade C Env, 4-2.J41 also harbors this conserved sequence (Figs. 1a, 3a, 4a). Previously, several studies described this conserved motif as hydrophilic and highly immunogenic [35, 36]. We verified it by Kyte and Doolittle analysis (data not shown). To investigate the role of this conserved hydrophilic domain (CHD) on the antigenicity/conformation of 4-2.J41 ectodomain, we constructed a series of CT mutants (Fig. 4a). Proteins expressed from these constructs retained their ability to bind to bNAb, VRCO1 similar to 4-2.J41_WT_ Env (Fig. 4b). The mutant protein expressed from the construct, 4-2.J41ΔCT_736_, which contains 12 amino acids of CHD, showed enhanced binding to non-NAb, F105 (Fig. 4b) similar to that of 4-2.J41ΔCT_711_ and 4-2.J41ΔCT_716_ Envs. The cytoplasmic tail truncated mutant Env, 4-2.J41ΔCT_744_ also showed moderate increase in binding to non-NAb, F105 (Fig. 4b). The mutants truncated at aa positions, 746, 747, 749, 752 and 754 showed minimal binding to F105, comparable to 4-2.J41_WT_ Env (Fig. 4b). In order to determine whether native conformation of the CHD mutant Env proteins is maintained, we also studied the binding of conformation dependent non-neutralizing antibodies, b6 and 17b to these mutant Envs (Fig. 4c). Truncating the CT at positions 737 (4-2.J41ΔCT_736_) and 745 (4-2.J41ΔCT_744_) resulted in partial deletion of the CHD whereas truncations at positions 746 (4-2.J41ΔCT_745_), 747 (4-2.J41ΔCT_746_), and 749 (4-2.J41ΔCT_748_) restored the CHD domain. Restoring the CT to amino acid 746 resulted in reduction in binding to the non-NAbs b6 and 17b comparable to wild type 4-2.J41 but best results were obtained with the construct 4-2.J41ΔCT_753_ (Fig. 4c). Taken together, our results suggest that the presence of the entire CHD, which ends at position 745 of the CT of 4-2.J41 Env is sufficient for restoring the native-like ectodomain conformation in terms of differential binding to VRCO1 and F105 and marginal binding to non-NAbs, b6 and 17b, similar to the wild type protein.

### Ectodomain properties of 4-2.J41ΔCT_753_ Env

**Fig. 5:**
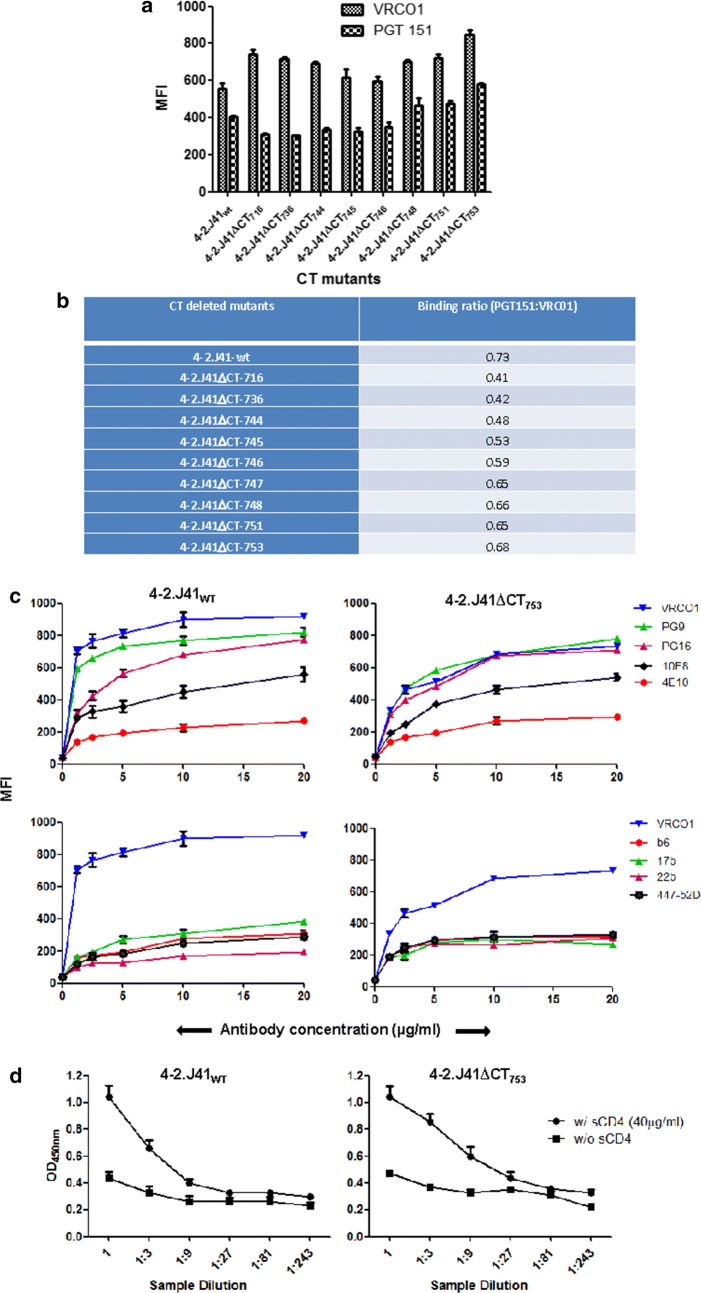
Restoration of conformation/antigenicity in 4-2.J41_753_ Env. **a** Binding of CT-deleted mutant Envs expressed on 293T cells to bNAbs, PGT151 (trimer-selective, cleavage-specific) and VRCO1 (cleavage non-specific) at 20 µg/ml antibody concentration by FACS based cell surface staining assay. The error bars indicate the standard mean obtained from two independent experiments run in duplicates, *P* < 0.0001. **b** Ratio of binding to PGT151 versus VRC01 antibodies by different CT deleted mutants in cell-surface binding assay. **c** Binding of 4-2.J41ΔCT_753_ mutant (upper panel) to CD4bs-directed bNAb, VRC01, glycan directed, V1/V2 conformation-dependent bNAbs, PG9 and PG16; gp41-directed bNAbs, 10E8 and 4E10 over a range of concentrations. Binding of 4-2.J41ΔCT_753_ mutant to non-NAbs, b6, 17b, 2.2B and 447-52D (lower panel). 4-2.J41_WT_ was used as positive control. **d** ELISA to determine the sCD4-induced gp120 shedding from 4-2.J41ΔCT_753_ Env transfected 293T cells incubated with and without sCD4 (40 µg/ml). 4-2.J41_WT_ Env was used as positive control

We next sought to determine whether the CHD can restore trimerization property similar to 4-2.J41_WT_ Env. We determined the binding of different ΔCT mutant Envs to the cleavage-specific, trimer-selective bNAb, PGT151 [37] at a concentration of 20 µg/ml (Fig. 5a). As shown in Fig. 5a, b, if binding to the cleavage-non-specific bNAb VRCO1 is taken as a measurement of expression of both cleaved and uncleaved Envs on the cell surface, CT truncated Envs show decreased binding to PGT151. The partial or complete deletion of CHD at positions 717 (4-2.J41ΔCT_716_), 737 (4-2.J41ΔCT_736_) and 745 (4-2.J41ΔCT_745_) resulted in the mutants showing a binding ratio (PGT151:VRCO1) of 0.41, 0.42 and 0.48, respectively as compared to the ratio of 0.73 for full length 4-2.J41 (Fig. 5b). However, after restoring the complete CHD, the binding ratio of Env 4-2.J41ΔCT_753_ increased to 0.68 (Fig. 5b).

We further investigated the binding of 4-2.J41ΔCT_753_ Env to bNAbs PGT121, PG9, PG16, and gp41 specific mAbs, 4E10, 10E8 (Fig. 5c). The bNAbs, PGT121, PG9 and PG16 are N-glycan dependent antibodies, of which PGT121 binds to monomeric gp120 and the others bind to quaternary epitopes. 4-2.J41ΔCT_753_ Env binds to PGT121, PG9, PG16 and 10E8 similar to the wild type protein (Fig. 5c). However, the binding of 4-2.J41ΔCT_753_ Env to 4E10 was minimal (Fig. 5c). Next, we examined the binding ability of 4-2.J41ΔCT_753_ Env to different non-NAbs (Fig. 5c). The non-NAbs, b6, 17b, 2.2B and 447-52D binds poorly to 4-2.J41ΔCT_753_ and 4-2.J41_WT_ Envs (Fig. 5c). Overall, these results demonstrate that restoring the CT of 4-2.J41 up to position 753 containing the CHD domain restores ectodomain antigenicity and conformation similar to that of 4-2.J41_WT_. We further compared the cleavability of 4-2.J41_WT_ and 4-2.J41ΔCT_753_ by studying the CD4 induced gp120 shedding of 4-2.J41ΔCT_753_ with full length 4-2.J41 Env in the absence and presence of 40 ug/ml concentration of sCD4 (Fig. 5d). We found by ELISA assay that the shedding of gp120 from both 4-2.J41_WT_ and 4-2.J41ΔCT_753_ is comparable and this shedding is enhanced upon incubation with sCD4 at the concentration of 40 ug/ml CD4 (Fig. 5d) suggesting that 4-2.J41ΔCT_753_ Env is cleaved to a similar extent as the wild type protein.

### Effect of CT truncation on three other efficiently cleaved Envs

**Fig. 6:**
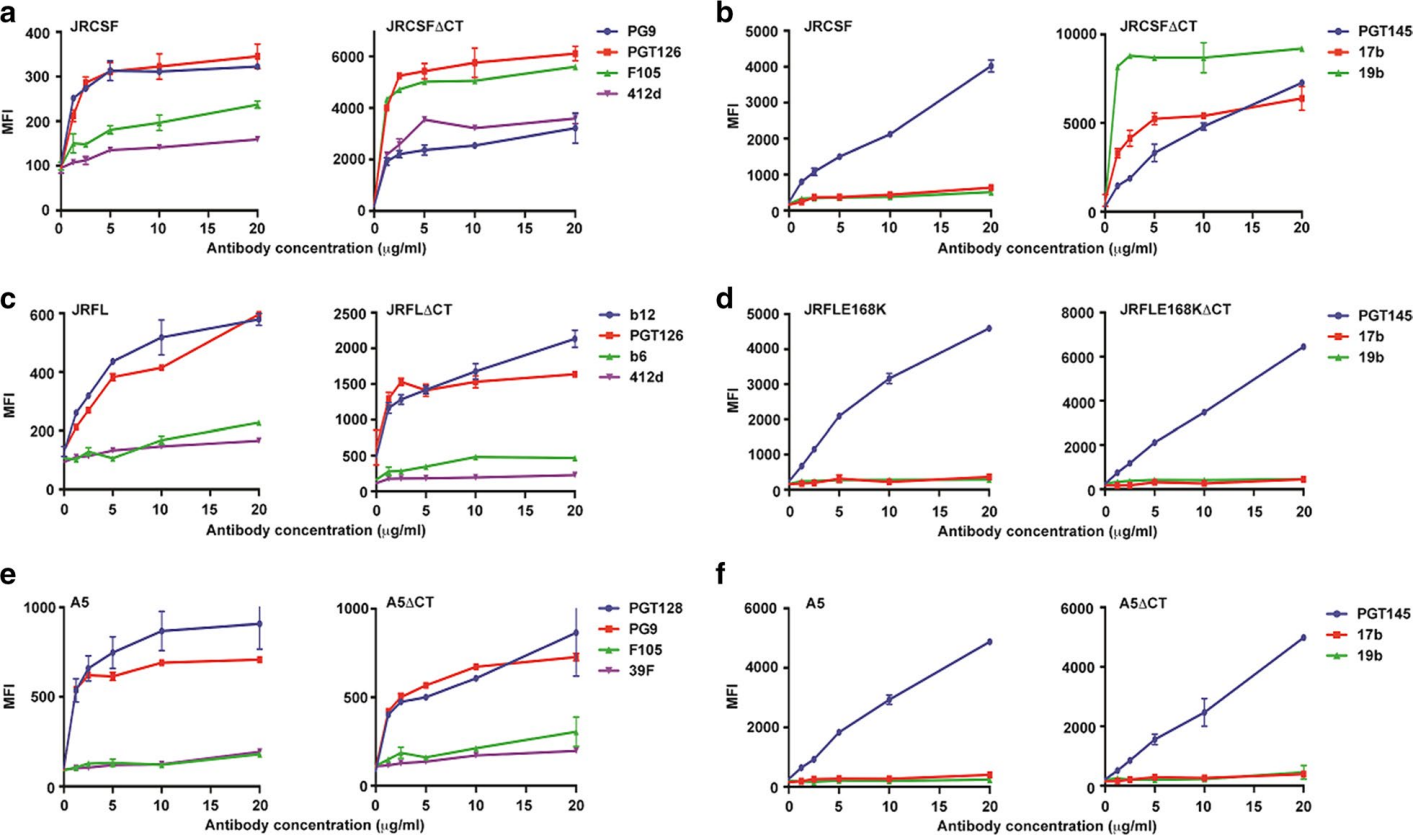
CT-deleted JRCSF but not JRFL and A5, have altered cell surface antigenicity/conformation**. a**, **c**, **e** FACS based cell surface staining assays of JRCSF, JRCSFΔCT with bNAbs, PGT126, PG9 and non-NAbs, F105, 412d; JRFL, JRFLΔCT with bNAbs, b12, PGT126 and non-NAbs, b6 and 412d; A5, A5ΔCT with bNAbs, PGT128 and PG9 and non-NAbs, F105, 39F over a range of antibody concentrations. **b**, **d**, **f** FACS based cell surface staining assays of JRFLE168K, JRFLE168KΔCT, A5, A5ΔCT, JRCSF, JRCSFΔCT with trimer-selective, cleavage-specific bNAb, PGT145 and non-NAbs, 17b (CD4i) and 19b (V3) over a range of antibody concentrations

Our data with 4-2.J41 Env clearly demonstrated that CT truncation alters ectodomain (ET) conformation/antigenicity which is restored similar to the wild type protein by restoring the conserved hydrophilic domain at its C-terminus. Next, we checked for ectodomain conformation/antigenic integrity upon CT truncation of the efficiently cleaved clade B Envs, JRFL [8] and JRCSF [21] and the clade A Env, A5 [19]. We first investigated the effect of C-terminal deletion on the cell surface expression, conformation and antigenicity of JRFL, JRCSF, and A5. As shown in Fig. 6a–f, deletion of the C-terminus resulted in higher expression of JRFLΔCT and JRCSFΔCT as the cleavage-independent bNAbs b12, PG9 and PGT126 showed much higher binding to ΔCT forms as compared to wild-type but due to some unknown reasons, no difference in expression was observed between A5 and A5ΔCT as the cleavage-independent bNAbs, PG9 and PGT128 showed similar binding to both forms of Env (Fig. 6e). The major endocytosis signals GYXXϕ and LL in the C-terminus of Env required for its internalization and whose removal or mutation increases Env expression on cell surface [38, 39] are both conserved in A5 (Additional file 2: Fig. S1a). Neither any toxicity nor any change in viability or toxicity induced changes in morphology of 293T cells transfected with plasmid expressing A5ΔCT Env was observed (Additional file 2: Fig. S1b). JRCSFΔCT Env binds to non-NAb F105 as efficiently as the bNAb, PGT126. Furthermore, the binding of JRCSFΔCT Env to the conformational bNAb, PG9 is reduced to similar to the level of non-NAb, 412d. All these data suggest that the exposure of certain non-neutralizing epitopes in JRCSFΔCT is due to the conformational change after deletion of the CT domain of the Env (Fig. 6a). However, both JRFLΔCT and A5ΔCT showed only marginal binding to non-NAbs, b6, 412d and F105, 39F (Fig. 6c, e), respectively. Due to the lack of presence of epitopes for certain neutralizing antibodies in different Envs, we used a combination of different antibodies in Fig. 6a, c, e. In order to directly compare the effect of CT deletion of JRFL, JRCSF and A5 on its binding to bNAbs and non-NAbs and also to investigate whether the CD4i and immunodominant V3 epitopes are exposed in ΔCT versions of these Envs, we studied their binding to the bNAb, PGT145 and non-NAbs, 17b and 19b (Fig. 6b, d, f). For this purpose, we used the JRFL mutant JRFLE168K as JRFL by itself does not bind PGT145. As shown in Fig. 6d, f, JRFLE168K and A5 and their ΔCT counterparts bind to PGT145 efficiently but weakly to 17b and 19b. However, JRCSFΔCT binds more efficiently to the non-NAbs, 17b and 19b as compared to PGT145 (Fig. 6b) suggesting that the immunodominant epitopes, CD4i and V3 are exposed in JRCSFΔCT. Thus, the homologous Envs, JRFL and JRCSF, isolated from the same patient, showed opposite antigenic phenotype upon deletion of the C-terminus, indicating that the CT of Envs may regulate conformation and antigenicity differently. Results from all these studies suggest that the CT of individual efficiently cleaved, functional Envs has varied effects on their expression, conformation and antigenicity.

### JRFLΔCT, JRCSFΔCT and A5ΔCT are efficiently cleaved

We next investigated whether the observed alteration in conformation/antigenicity of JRCSFΔCT was due to inefficient cleavage of the ΔCT version. The trimer selective, cleavage specific bNAbs, PGT151 and PGT145 show significant reduction in binding to uncleaved forms (cleavage site is mutated from REKR to SEKS) of the efficiently cleaved Envs, JRFL, JRCSF, 4-2.J41 and A5 [8, 20, 21]. Thus, efficient binding to these bNAbs is a good measure of cleavage efficiency. The Envs JRFLE168K, JRCSF, A5 and their ΔCT variants bind efficiently to bNAb, PGT145 (Fig. 6b, d, f) suggesting that C-terminal truncation does not affect cleavage efficiency. We further verified that the ΔCT forms of these Envs are efficiently cleaved by immunoprecipitation of plasma membrane (PM) fractions of transfected cells using cleavage independent bNAbs, VRC01 and PG9 (Additional file 3: Fig. S2). We used JRFL as a control as it shows only gp120 band from precipitates of biotinylated cell surface fractions of transfected cells and is therefore efficiently cleaved [8]. Previously using uncleaved (gp160 only band) and partially cleaved (both gp120 and gp160 bands) Envs as controls, we have shown that VRC01-immunoprecipitates of PM fraction of JRFL transfected cells gives only gp120 band in western blot [19, 21]. Western blot analysis of the immunoprecipitates of JRFL with VRC01 used as control, and JRFLΔCT, JRCSFΔCT, 4-2.J41ΔCT and A5ΔCT with either VRC01 or PG9 shows gp120 band, with minor size variations possibly due to differential glycosylation (Additional file 3: Fig. S2). Taken together our studies strongly suggest that C-terminal deletion of efficiently cleaved Envs JRFL, JRCSF and A5 do not affect their cleavage efficiency.

### Conserved hydrophilic domain in CT of JRCSFΔCT_759_ restores wild type conformation/antigenicity

**Fig. 7:**
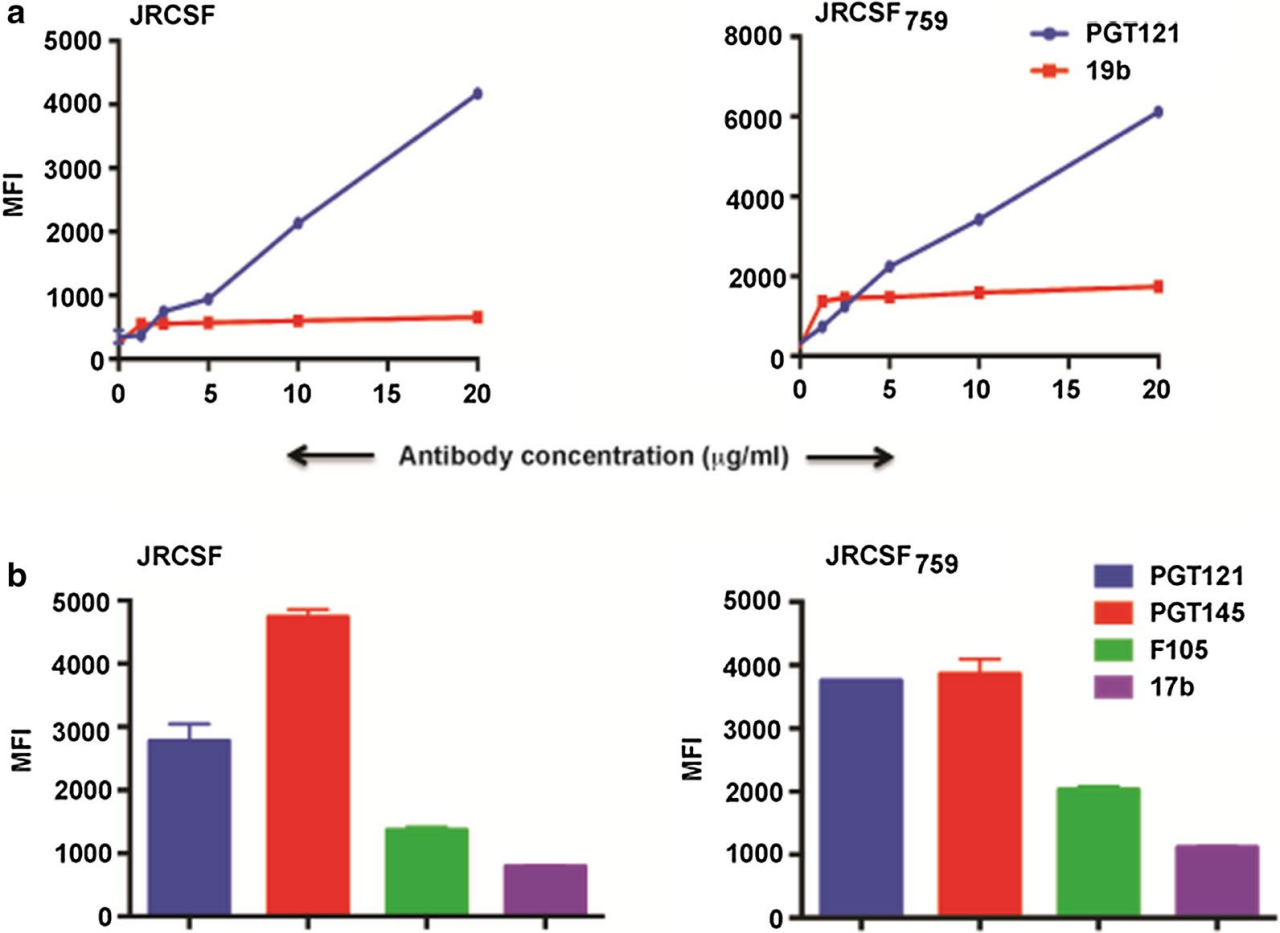
Restoration of conformation/antigenicity in JRCSFΔCT_759_ Env. **a** FACS based cell surface staining assays of JRCSF and JRCSFΔCT_759_ with bNAb, PGT121 and non-NAb, 19b over a range of antibody concentration. **b** FACS based cell surface staining assays of JRCSF and JRCSFΔCT_759_ with bNAbs, PGT121, PGT145 and non-NAbs, F105 and 17b at 20 μg/ml antibody concentration

Next, we tested whether the region corresponding to the CT of 4-2.J41ΔCT_753_, containing the conserved hydrophilic domain, in JRCSF was able to restore wild type antigenicity and conformation. As 4-2.J41ΔCT_759_ showed the best result in terms of restoring native conformation and antigenicity we tested JRCSFΔCT_759_ for its ability to bind the bNAbs PGT121 (JRCSF does not bind efficiently to VRC01) and 2G12 (data not shown) and non-NAb 19b and compared it with JRCSF wild type (Fig. 7a). JRCSF_WT_ binds to PGT121 and 2G12 efficiently but weakly to 19b, and JRCSFΔCT_759_ was able to restore wild type properties as it binds efficiently to PGT121 and 2G12 but marginally to 19b (Fig. 7a). Similarly JRCSFΔCT_759_ showed efficient binding to the trimer-selective, cleavage-specific bNAb PGT145 and weaker binding to the non-NAbs F105 and 17b (Fig. 7b) suggesting restoration of wild type properties. Taken together these studies suggest that the conserved hydrophilic domain containing region in JRCSFΔCT_759_ restores wild type conformation and antigenicity similar to 4-2.J41ΔCT_753_.

### Determination of infectivity and antigenicity of cytoplasmic deleted mutant viruses

**Fig. 8:**
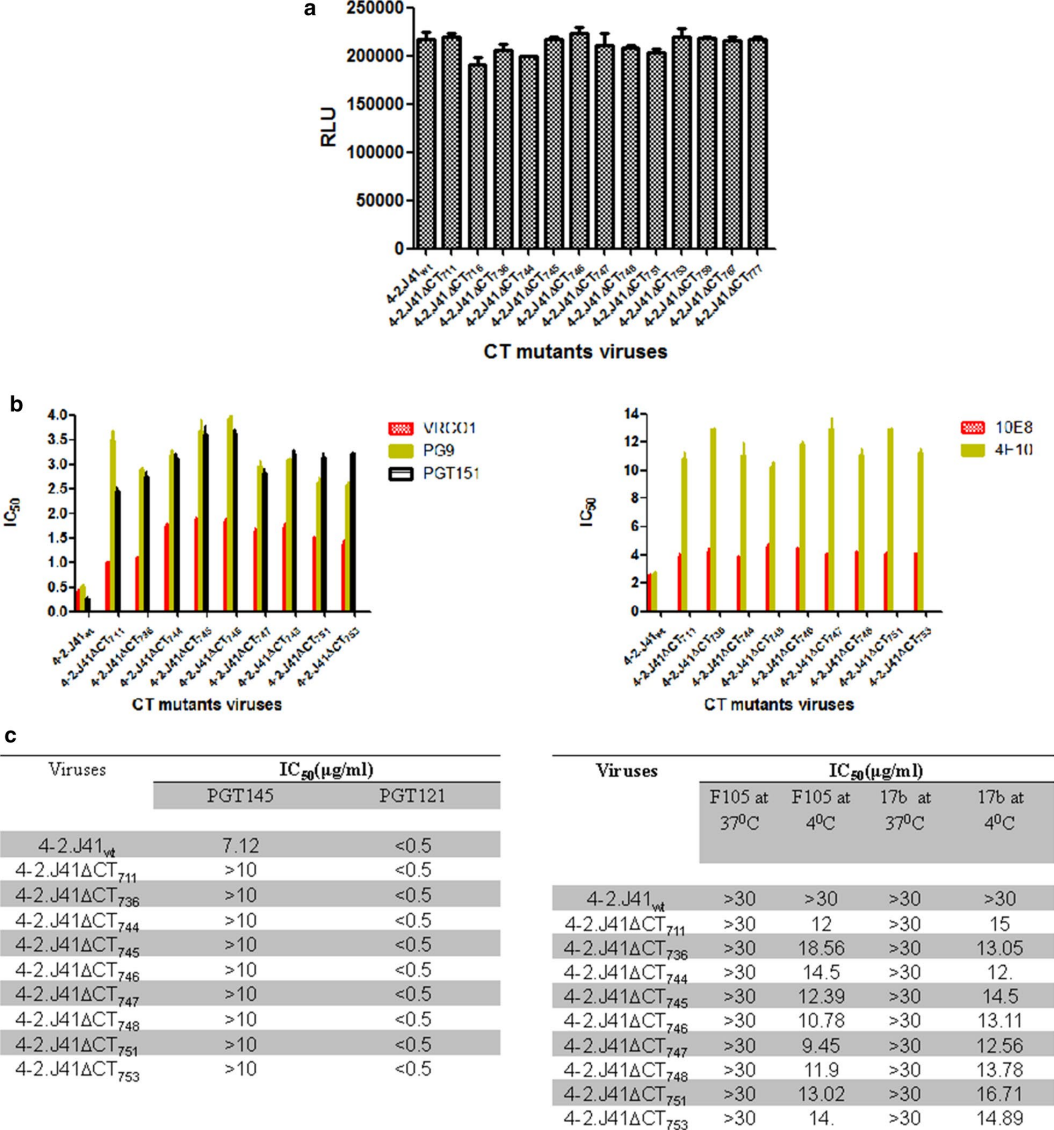
Infectivity and neutralization assays of 4-2.J41 mutant Env pseudotyped viruses. **a** Infectivity of 4-2.J41 Env wild type and deletion mutant pseudoviruses using TZM-bl reporter based cell assay. **b** IC_50_ values of 4-2.J41 wild type and deletion mutant Env pseudotyped viruses with the cleavage non-specific bNAb VRC01, glycan-dependent and conformational bNAb, PG9, trimer-selective and cleavage-specific bNAb, PGT151, MPER-directed bNAbs, 10E8 and 4E10. **c** IC_50_ values of 4-2.J41 wild type and deletion mutant Env pseudotyped viruses with bNAbs, PGT121 and PGT145 (left panel) and non-NAbs F105 and 17b at 37 and 4 °C (right panel)

To determine the effect of CT deletion on infectivity and neutralization sensitivity of 4-2.J41 viruses, we produced different 4-2.J41 CT-deleted Env pseudotyped viruses by transfection of 293T cells with pSG3Δenv vector backbone and Env plasmids [40]. Infectivity of the pseudotyped viruses was determined using TZM-bl reporter cells [52] by infecting with supernatants containing equal amounts of viral particles as measured by p24 ELISA (normalized to p24) and tested for luciferase activity after 48 h. We consistently found that all CT-deleted mutant Env pseudotyped viruses show high luciferase titers (Fig. 8a, *P* > 0.05) similar to that of wild type virus. Our results suggest that all the CT mutants are capable of viral entry and replication in TZM-bl cells. We further assessed the neutralization sensitivity of CT-deleted mutants to various monoclonal antibodies as described in Materials and Methods and determined the IC_50_ values (µg/ml). Among the various neutralizing mAbs we tested, all the CT-deleted mutants were highly sensitive to VRCO1 (anti-CDbs) antibody with IC_50_ values < 2 (Fig. 8b). In case of mAbs PG9 (anti-V2/V3) and PGT151 (cleavage and trimer specific), although the IC_50_’s are < 4, the CT mutant viruses show a 5-10 fold decrease in neutralization sensitivity as compared to wild type virus (Fig. 8b). Moreover, mAbs PGT121 and 10E8 affected the CT deleted mutants similarly (Fig. 8b, c), whereas the CT mutants showed more resistance to PGT145 (cleavage, quaternary specific) and 4E10 (MPER directed) mAb (Fig. 8b, c). The CT deleted mutant viruses showed neutralization resistance to non-NAbs (F105, 17b) with IC_50_ values > 30 when antibody was incubated with the viruses at 37 °C followed by TZM-bl assay. However, when incubation was carried out at 4 °C, the CT deleted mutant viruses were less resistant to non-NAbs (Fig. 8c, *P* < 0.0001). Our results suggest that at the virus level deletion of the CT of 4-2.J41 had no effect on infectivity and modest effects on neutralization sensitivity to bNAbs and non-NAbs. The conserved hydrophilic domain did not appear to restore the modest effects on neutralization sensitivity in 4-2.J41ΔCT_711_ Env to wild type levels. With JRCSFΔCT Env pseudotyped viruses, the neutralization sensitivity (IC_50_ values) with the bNAbs VRC01, PG9, PGT121, PGT151 and PGT145 was very similar to the wild type protein (Additional file 4: Fig. S3). Similar to wild type viruses, JRCSFΔCT Env pseudotyped viruses were resistant to neutralization by the non-NAbs, F105 and 17b (Additional file 4: Fig S3). About a 5 fold higher sensitivity was observed for neutralization by 10E8 (MPER-directed bNAb) for JRCSFΔCT Env pseudoytped virus as compared to JRCSF Env pseudotyped virus (Additional file 4: Fig S3). Thus, these studies suggest that sensitivity of JRCSFΔCT pseudotyped viruses to various bNAbs and non-NAbs is not significantly different from that of virus pseudotyped with full length JRCSF Env.

## Discussion

Several functions of Env are regulated by its C-terminal tail (CT) [30, 41, 42]. The unusually long conserved CT in HIV-1 Env mediates native conformation, and various Env functions, including trafficking, proper surface expression, membrane fusion, virion replication and budding [25, 26, 34, 43–45]. Among these functions, the effect of CT on expression, conformation and antigenicity are critical for developing Env-based immunogens. Studies on HIV-1 and SIV Envs have demonstrated that truncation of the CT alters the conformation of the ectodomain but the exact mechanism is poorly understood [29–31]. Previously, it has been suggested, that for partially cleaved Envs, removal of the C-terminus results in increase in expression and the exposure of epitopes for non-neutralizing antibodies [32]. In addition, CT truncation have been reported to affect binding to conformational mAbs, CD4bs-targeting and CD4i-directed antibodies in comparison to full-length protein [30] although the cleavage status of these Envs has not been reported.

Efficient cleavage of gp160 Envs into their constituent subunits gp120-gp41 is a pre-requisite for selective exposure of bNAb-binding epitopes and is thus the closest mimic of native, functional Envs – a property desirable for immunogen development. The CT of Envs is often deleted in order to enhance the expression and also to stabilize the soluble form of native-like trimeric Env immunogens [11, 14, 28]. The identification of the four efficiently cleaved Envs (JRFL, JRCSF, 4-2.J41 and A5) from clades A, B and C [8, 20, 21] allowed us to analyze the effect of deleting the C-terminus on the expression, conformation and antigenicity of these cross clade Envs, parameters important for designing immunogens for DNA, viral vector, virus-like particle as well as soluble protein based vaccination. We find that CT-deleted mutants of efficiently cleaved Envs display phenotypic diversity. While the expression of JRFL, JRCSF and 4-2.J41 is increased, the expression of A5 does not significantly change even though it retains the two major endocytosis signals in its CT. Conformation and antigenicity of C-terminus deleted JRFL and A5 does not change but in the case of JRCSF and 4-2.J41, non-neutralizing epitopes get exposed upon deletion of CT. We also find that the homologues JRFL and JRCSF behave differently when their CT is deleted.

The precursor gp160 polypeptide of HIV-1 Env is synthesized in ER and transported to the Golgi/*trans*-Golgi network for post translational modification and cleavage to attain native conformation and thus generate efficiently cleaved, functional Envs [44, 46]. It is possible that JRCSFΔCT and 4-2.J41ΔCT Env is inefficiently cleaved, which exposes epitopes for non-NAbs. However, our data demonstrates that there is no significant reduction in cleavage of ΔCT forms of efficiently cleaved Envs. This led us to investigate the role of different domains and regions in the CT of 4-2.J41 and JRCSF that may be required for maintaining native-like ectodomain conformation/antigenicity of the wild type proteins. The role of different conserved domains in the CT of Env in its functions is not fully understood. In this study, we have used deletion constructs and cell surface staining to map the conserved domain(s) in the CT of 4-2.J41 that can restore wild type antigenicity/conformation. The CT of 4-2.J41 Env contains three conserved domains called lentivirus lytic peptides (LLP2, LLP3, and LLP1) that show high degree of conservation among CTs of different HIV-1 strains and also among CTs of other lentivirus Envs such as HIV-2 and SIV [24, 34, 47]. Deletion of LLP domains in CT of 4-2.J41 did not alter the integrity of its ET. We evaluated the role of another highly conserved hydrophilic domain [22, 48] in restoring the integrity of 4-2.J41 Env and JRCSF Env ET [24, 35, 42]. Using in silico analysis, we confirmed the high hydrophilicity of this conserved region in the CT of 4-2.J41 Env between amino acids 724-745 and we termed it as conserved hydrophilic domain (CHD). Restoration of the CHD-containing region in 4-2.J41ΔCT_753_ restored the native, wild-type conformation and antigenicity of ET. 4-2.J41ΔCT_753_ Env containing the CHD was sufficient to restore conformation of the ectodomain as evaluated by efficient binding to VI/V2 quaternary epitope targeted mAbs, PG9 and PG16, MPER-directed mAbs, 4E10 and 10E8 and marginal binding to several non-NAbs. Similarly, restoration of the conserved hydrophilic region at the C-terminus in JRCSFΔCT_759_ restored the ET conformation/antigenicity to wild type protein. To the best of our knowledge a role for the conserved hydrophilic domain in Env ectodomain conformation and antigenicity has not been previously demonstrated. Taken together these studies for the first time demonstrates a role for the conserved hydrophilic domain in the C-terminal tail of a subset of functional Envs in maintaining their ectodomain conformation/antigenicity. Furthermore, 4-2.J41 CT-deleted Env pseudotyped virus particles showed only modest neutralization sensitivity variation to different bNAbs and modest temperature dependent sensitivity to non-NAbs. These effects were not restored to wild type levels by restoring the conserved hydrophilic domain in 4-2.J41ΔCT_753_ Env pseudotyped viruses. The neutralization sensitivity of JRCSF and JRCSFΔCT pseudotyped viruses to different bNAbs and non-NAbs were found to be similar with an exception for 10E8. Thus, these results suggest that effect of the conserved hydrophilic domain in regulating the antigenicity and conformation of 4-2.J41 and JRCSF Envs is limited to the cell surface expressed protein. Based on these results we hypothesize that deletion of the CT in 4-2.J41 and JRCSF Envs affects their native structure which leads to the generation of a heterogeneous population of Envs like native, near-native and non-native molecules within the cell but in the presence of one or more viral protein(s) only the native or near-native ΔCT Envs are incorporated into viral particles. Viral and cellular factors play important roles in HIV-1 particle assembly [44]. Gag plays a critical role in this process and MA (matrix) directs incorporation of Env into virions during assembly [44]. Two of the proposed Env incorporation into virion models posits that Env is taken up in assembling viruses through direct association or a host protein-mediated interaction with Gag [44]. Thus, there may be a mechanism during the assembly of viral particles to preferentially incorporate functional Envs. Some degree of incorporation of non-native forms can be seen in these cell lines e.g. incorporation of small amounts of uncleaved Envs in virus-like particles [28]. The conserved hydrophilic domain, also called Kennedy epitope, has been proposed to be intracytoplasmic in the traditional model while in the alternate model this region is suggested to be extracellular [48]. However, the NMR structure of the region containing the conserved hydrophilic domain from the pNL4-3 isolate shows that it is unstructured and not associated with membrane [49] although it is still possible that the characteristics of this domain is strain-specific. Furthermore, in the context of the virus, the CHD alone may not be sufficient to restore wild type properties as it is evident by the inability of 4-2.J41ΔCT_753_ Env pseudotyped viruses to maintain the neutralization sensitivity like the 4-2.J41 Env pseudotyped virus. Therefore, how and why this domain determines ET integrity in only some but not all efficiently cleaved Envs need to be investigated. Finally, including the conserved hydrophilic domain in Env-based soluble immunogen design may be necessary for Envs whose CT regulate their ET antigenicity and conformation on the plasma membrane.

It is to be noted here that we have looked at the effect of CT deletion on efficiently cleaved Envs in the permissive cell line 293T as this cell line and its derivatives are frequently used to produce Env-based immunogens. Furthermore, the ΔCT Envs are efficiently incorporated into viral particles in this cell line and thus making it easier to compare the neutralization sensitivity against a number of antibodies to virus pseudotyped with full length Env. Furthermore, most of the assays used for this study are well established in 293T cell line. Besides from a vaccine immunogen development point of view, study in 293T cells or their derivatives may be relevant. However, T cells, peripheral blood mononuclear cells (PBMCs) and monocyte-derived macrophages are the natural targets for HIV-1 infection and the CT of Env is required for spreading productive infection in these cell types [50]. So in order to better understand the biological role of the CT and the conserved hydrophilic domain of the efficiently cleaved Envs, used in this study, in determining Env ET antigenicity/conformation, it will be necessary to extend this study to these cell types in future.

## Conclusion

The long C-terminal tail of Env contains several domains and plays an important role in multiple functions of the protein including in maintaining its ectodomain antigenicity and conformation. Using four efficiently cleaved, functional Envs we show that deletion of the CT has variable effect on the ectodomain antigenicity and conformation with a subset represented by 4-2.J41 and JRCSF Envs being affected while the Envs JRFL and A5 remain unaffected. Using deletion analysis we find that a region in the CT containing a conserved hydrophilic domain restores native ectodomain antigenicity and conformation in 4-2.J41 and JRCSF. This effect is restricted to the plasma membrane bound Env and is not seen in Env on the viral membrane. This study demonstrates the importance of the conserved hydrophilic domain in maintaining the integrity of the ectodomain on the cell surface and will help further guide to design native-like functional Env-based immunogens.

## Methods

### Cell lines and antibodies

293T cells were purchased from the American Type Culture Collection (ATCC). TZM-bl cells were obtained from NIH AIDS Reagent Program. The cells were maintained in Dulbecco’s modified Eagle’s medium (DMEM) (Invitrogen, USA) supplemented with 10% fetal calf serum (HiMedia, USA), 20 mM l-glutamine, 100 U/ml penicillin, and 100 μg/ml streptomycin. The broadly neutralizing antibodies (VRC01, PGT121/128, PGT145, PGT151, PG9, PG16, 10E8 and 4E10) and non-neutralizing antibodies (F105, b6, 39F, 447-52D, 22b and 17b) were obtained from the IAVI Neutralizing Antibody Center (NAC) at TSRI, La Jolla, California. The anti-gp120 (clades A, B, C) rabbit polyclonal antibodies were purchased from ABLinc, USA.

### Plasmids and mutagenesis

The full-length Env 4-2.J41 (gp160) clones were obtained from National AIDS Research Institute, Pune, India and have been previously described [51]. To generate CT deleted constructs, stop codons were introduced into the cytoplasmic tail at amino acid (aa) position 712 (4-2.J41ΔCT_711_), 717 (4-2.J41ΔCT_716_), 737 (4-2.J41ΔCT_736_), 745 (4-2.J41ΔCT_744_), 746 (4-2.J41ΔCT_745_), 747 (4-2.J41ΔCT_746_), 749 (4-2.J41ΔCT_748_), 752 (4-2.J41ΔCT_751_), 754 (4-2.J41ΔCT_753_), 760 (4-2.J41ΔCT_759_), 768 (4-2.J41ΔCT_767_), 778 (4-2.J41ΔCT_777_) in pSVIII-env plasmid backbone by PCR amplification using *Phusion* polymerase following manufacturer’s protocol. The reaction mixtures were digested with *Dpn*1, transformed into competent cells and plated onto LB-ampicillin plates. The mutants were confirmed by sequencing of the plasmid DNA. JRCSFΔCT and JRCSFΔCT_759_ were generated by introducing stop codons as described above.

### Cell surface assay using FACS-based method

All flow cytometry-based cell surface assays were carried out as described previously [20]. 293T cells were transfected, harvested and then washed three times with FACS buffer I (DMEM + 5% HI-FBS). Cells were stained with increasing concentration of different bNAbs and non-NAbs in 96 well U-bottomed plate wells for 1 h at room temperature (RT). The cells were then washed with FACS buffer I, followed by incubation with PE-conjugated goat anti-human secondary antibody (1:200 dilutions, Jackson ImmunoResearch) for 1 h. at RT. Next, the cells were washed with FACS buffer II (PBS + 5% HI-FBS) and fixed with 0.5% paraformaldehyde. Finally, the stained and fixed cells were analyzed in a FACS Canto analyzer (BD Biosciences) and MFI determined by using FlowJo software (version 10.0.6, Tree Star Inc.).

### Analysis of sCD4-induced gp120 shedding

Soluble CD4 induced (sCD4-induced) shedding of gp120 was assayed as previously described [5, 20]. Briefly, 293T cells transfected with different plasmids harboring wild type or mutant Envs were harvested and washed with FACS buffer and then incubated with or without 50 µg/ml sCD4 (NIH AIDS Reagent) for 1 h at 4 °C with intermittent mixing of samples. Cells were centrifuged and the supernatant subjected to ELISA as described previously [20]. Supernatants were also subjected to western blot analysis anti-clade C Env gp120 polyclonal antibody.

### Western blot analysis and cell surface protein biotinylation

293T cells were transiently transfected with Env constructs and 48 h post transfection whole cell extracts were isolated using lysis buffer, the lysed proteins were denatured, reduced, separated by 10% SDS-PAGE, and analyzed by Western blotting using a 1:200 dilution of anti-gp120 (clade C) rabbit polyclonal antibody.

For cell surface biotinylation, the methods were performed as described previously with few modifications [20]. Briefly, 293T cells were co-transfected with plasmids expressing with CT deleted or wild type Env and Tat proteins as described above. 36–48 h post transfection the cells were harvested and washed with PBS (pH 8). The cells were labeled with freshly prepared 2-5 mM biotin (EZ-link Sulfo-NHS-LC-Biotin, Thermo Scientific, and Cat. No. 21335) in PBS (pH 8) for 30 min at 4 °C, washed with PBS twice, incubated with 50 mM glycine in PBS (pH 7.5) for 30 min at 4 °C with rotation and then washed twice with PBS. Cell pellets were resuspended in RIPA buffer (50 mM Tris-HCI pH 7.4, 25 ml of 1 M. 1% NP-40 5 ml. 0.5% Na-deoxycholate 2.5 g, 0.1% SDS, 150 mM NaCl, 2 mM EDTA containing protease inhibitors) and incubated on ice for 30 min. The cell extract was centrifuged at 13000 rpm for 30 min at 4 °C and the supernatant was subjected to precipitation with 100 µl slurry of high capacity neutravidin agarose (Thermo Scientific cat. no. 29204) (pre-washed with PBS) for 2 h at RT with rotation. Finally beads were washed three times with PBS + 1% Triton-X and washed beads were subjected to western blot analysis using rabbit anti-clade C antibodies as probes.

### p24 ELISA and HIV-1 infectivity assay (TZM-bl)

Mutant pseudotyped viruses were produced from 293T cells by transient transfection with Fugene (Promega,USA) as previously described [52]. The p24 values were measured using HIV-1 p24 Antigen Capture Kit (abcam USA) following the manufacturer’s instructions. Briefly, properly diluted virus samples were lysed and captured in a micro-ELISA plate at 37 °C for 1 h. The wells were then washed and the specifically captured p24 antigen was incubated with human anti-p24 polyclonal antibodies conjugated with peroxidase at 37 °C for 1 h. At the end of incubation, peroxidase substrate was added and the reaction was continued for 30 min at room temperature. The reaction was stopped by adding stop solution and OD read at 450 nm.

For determination of HIV-1 infectivity, briefly, TZM-bl cells were seeded in 96-well plates at 10^4^ cells per well in 100 µl complete DMEM and incubated for 24 h at 37 °C. Virus samples normalized to p24 value were added to cells in a total volume per well of 200 µl DMEM. Cells were harvested 48 h post-infection, and HIV-1 LTR-induced luciferase activity in the cells was determined using the Luciferase Assay System (Promega). Results are reported in relative luminescence units (RLU) as measured on a luminometer (PerkinElmer).

### Calculations and statistics

The Mean fluorescent intensity of a given experiment was calculated by finding average of duplicate wells and each experiment was repeated three times. Statistical analyses were performed using Prism, version 5.0, software (GraphPad Software). Error bars represent the standard errors of the mean (SEMs) of at least two independent experiments. Values were determined by Two-way ANOVA of pooled data.

## Additional files

**Additional file 1: Table S1a.** Ratio of binding to non-NAbs versus VRC01. **Table S1b.** Ratio of binding to bNAbs versus VRC01. **Table S1c.** Ratio of binding to PGT 151 versus VRC01.

**Additional file 2: Fig. S1.** Domains and endocytosis signals in JRFL, JRCSF, 4-2.J41 and A5. **(a)** Sequence comparisons of the C-terminal tails of 4-2.J41 (clade A), JRFL and JRCSF (clade B), A5 (clade A) and HxB2 showing that the endocytosis signals (highlighted in yellow) are largely conserved. Amino acid changes are marked in red. CHD (KE): Conserved Hydrophilic Region (Kennedy Epitope); LLP1, LLP2, LLP3 (Lentivirus Lytic Peptides 1,2,3). **(b)** Bright field image of A5 and A5ΔCT transfected whole cells.

**Additional file 3: Fig. S2.** Cleavage properties of ΔCT mutants of JRFL, JRCSF, 4-2.J41 and A5. Western blot analysis of immunoprecipitates (with cleavage non-specific bNAbs VRC01 and PG9) of plasma membrane fractions of JRFL, JRFLΔCT, JRCSFΔCT, 4-2.J41ΔCT, A5ΔCT transfected 293T cells using rabbit anti-clades A, B and C antibodies as probes.

**Additional file 4: Fig. S3.** Infectivity and neutralization assays of JRCSF and JRCSFΔCT pseudoviruses. **(a)** Infectivity of JRCSF Env wild type and JRCSFΔCT mutant pseudoviruses using TZM-bl reporter based cell assay. **(b)** IC_50_ values of JRCSF wild type and JRCSFΔCT mutant pseudoviruses with the cleavage non-specific bNAb VRC01, glycan-dependent and conformational bNAb PG9, PGT121, trimer-selective and cleavage-specific bNAbs PGT151 and PGT145, MPER-directed bNAbs 10E8 and non-NAbs F105 and 17b.

## Abbreviations

HIV-1: human immunodeficiency virus type I
Env: envelope
bNAb: broadly neutralizing antibody
non-NAb: non-neutralizing antibody
FACS: fluorescence activated cell sorting
PM: plasma membrane
IP: immunoprecipitation
CD4i: CD4 induced
CT: C-terminal tail
ET: ectodomain
