# Supplementary figures and images for: Cell surface ectodomain integrity of a subset of functional HIV-1 envelopes is dependent on a conserved hydrophilic domain containing region in their C-terminal tail
Source: Retrovirology. 2018 Jul 20;15:50. doi: 10.1186/s12977-018-0431-4 (PMC6053805; doi:10.1186/s12977-018-0431-4)

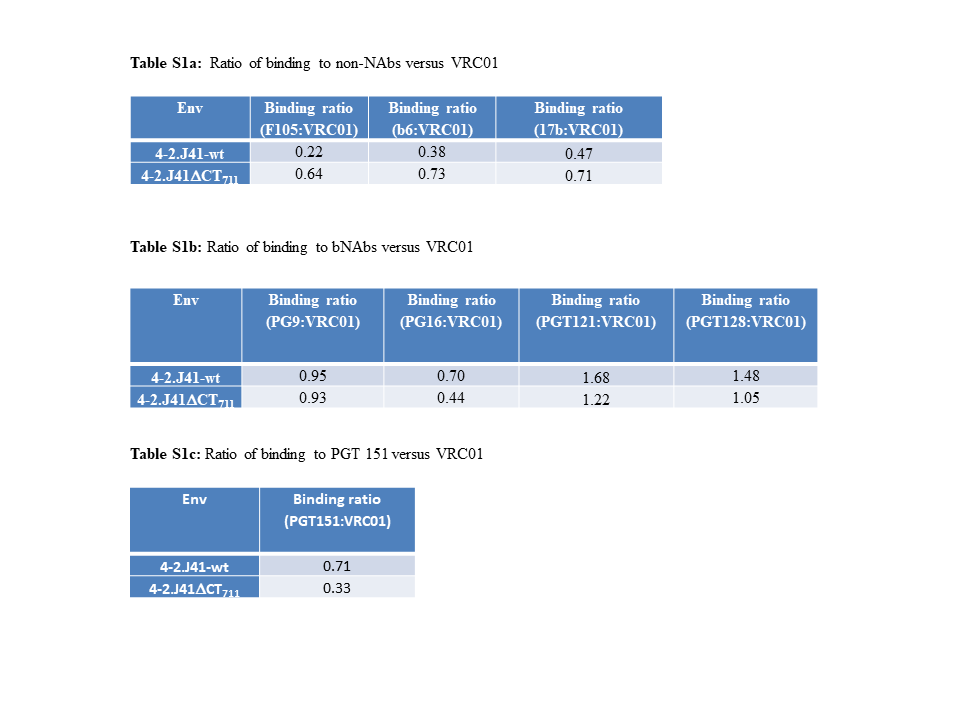

Supplement: Supplementary file 1 — Additional file 1: Table S1a. Ratio of binding to non-NAbs versus VRC01. Table S1b. Ratio of binding to bNAbs versus VRC01. Table S1c. Ratio of binding to PGT 151 versus VRC01. [file 12977_2018_431_MOESM1_ESM.tif]

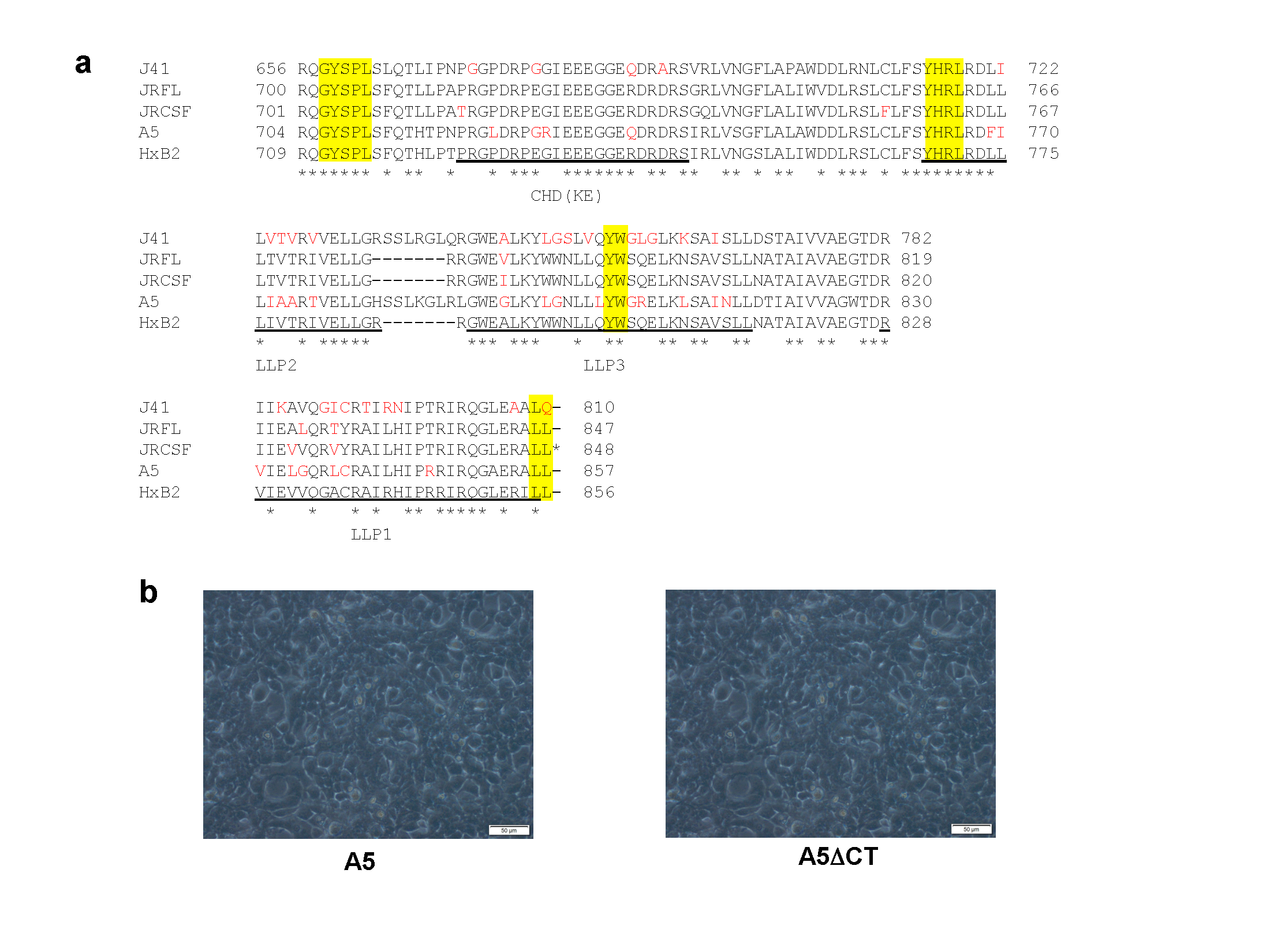

Supplement: Supplementary file 2 — Additional file 2: Fig. S1. Domains and endocytosis signals in JRFL, JRCSF, 4-2.J41 and A5. (a) Sequence comparisons of the C-terminal tails of 4-2.J41 (clade A), JRFL and JRCSF (clade B), A5 (clade A) and HxB2 showing that the endocytosis signals (highlighted in yellow) are largely conserved. Amino acid changes are marked in red. CHD (KE): Conserved Hydrophilic Region (Kennedy Epitope); LLP1, LLP2, LLP3 (Lentivirus Lytic Peptides 1,2,3). (b) Bright field image of A5 and A5ΔCT transfected whole cells. [file 12977_2018_431_MOESM2_ESM.tif]

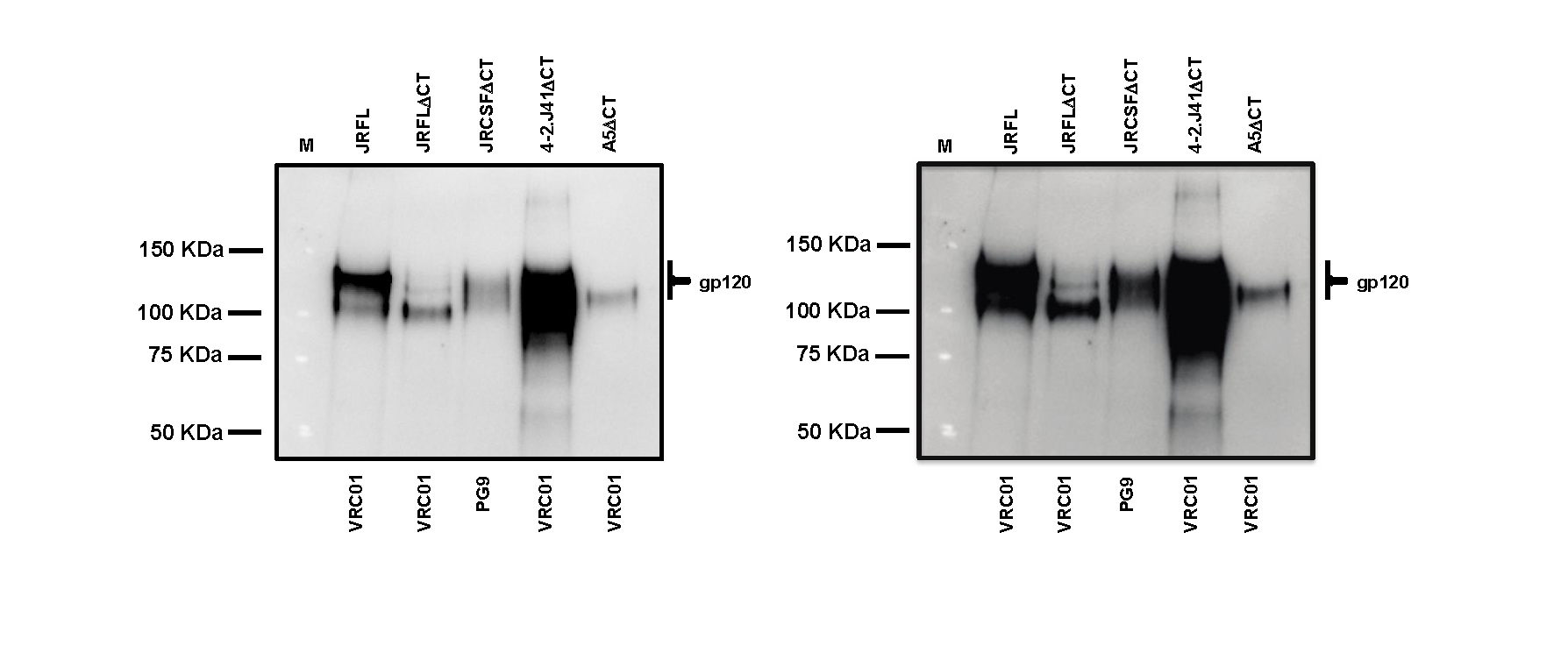

Supplement: Supplementary file 3 — Additional file 3: Fig. S2. Cleavage properties of ΔCT mutants of JRFL, JRCSF, 4-2.J41 and A5. Western blot analysis of immunoprecipitates (with cleavage non-specific bNAbs VRC01 and PG9) of plasma membrane fractions of JRFL, JRFLΔCT, JRCSFΔCT, 4-2.J41ΔCT, A5ΔCT transfected 293T cells using rabbit anti-clades A, B and C antibodies as probes. [file 12977_2018_431_MOESM3_ESM.tif]

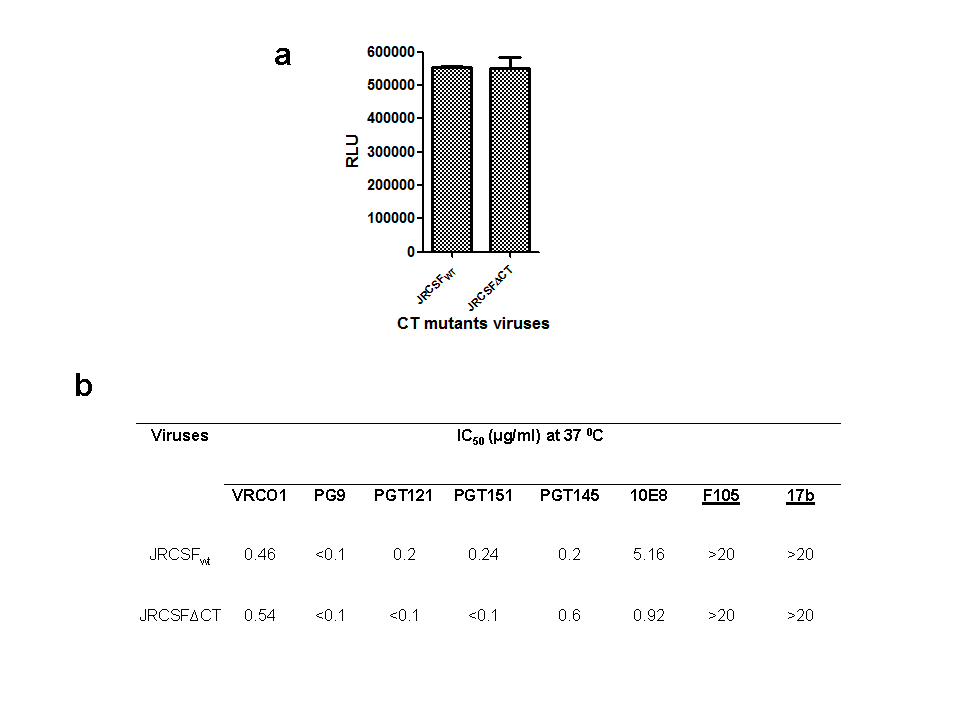

Supplement: Supplementary file 4 — Additional file 4: Fig. S3. Infectivity and neutralization assays of JRCSF and JRCSFΔCT pseudoviruses. (a) Infectivity of JRCSF Env wild type and JRCSFΔCT mutant pseudoviruses using TZM-bl reporter based cell assay. (b) IC50 values of JRCSF wild type and JRCSFΔCT mutant pseudoviruses with the cleavage non-specific bNAb VRC01, glycan-dependent and conformational bNAb PG9, PGT121, trimer-selective and cleavage-specific bNAbs PGT151 and PGT145, MPER-directed bNAbs 10E8 and non-NAbs F105 and 17b. [file 12977_2018_431_MOESM4_ESM.tif]
